# Geographic variation in the utilisation of specialist healthcare for patients with substance use disorders in Norway: a population-based registry study

**DOI:** 10.1007/s43999-025-00084-y

**Published:** 2026-01-05

**Authors:** Haji Kedir Bedane, Lars Lien, Jørgen G. Bramness, Per Arne Holman

**Affiliations:** 1 Research and Innovation Department, Førde Health Trust, Forde, Norway; 2 Research Center for Substance Use Disorders and Mental Illness, Innlandet Hospital Trust, Hamar, Norway; 3 Faculty of Health and Social Science, University of Inland Norway, Elverum, Norway; 4 Institute of Clinical Medicine, UiT – The Arctic University of Norway, Tromsø, Norway; 5 Section for Clinical Addiction Research, Oslo University Hospital, Oslo, Norway; 6 Department of Patient Safety and Research, Lovisenberg Diaconal Hospital, Oslo, Norway

**Keywords:** Geographic variation, Unwarranted variation, Health atlas, Substance use disorders (SUD), Mental healthcare, Patient rates, Treatment rates

## Abstract

**Purpose:**

The purpose of this study is to analyse geographic variation in rates of patients and service utilisation for persons with substance use disorders (SUD) across Norwegian hospital catchment areas from 2017 to 2021, considering both outpatient and Inpatient care across substance use diagnosis.

**Method and material:**

This registry-based study used data from the Norwegian Patient Registry and Statistics Norway, covering 58,889 unique patients and 121,495 patient-years. Adjusted for age and sex this material yields a national SUD treatment rate of 14.0 per 1,000 over five years, on average 5.8 per year with a declining annual rate from 6.1–5.5. Analyses included diagnoses related to alcohol, opioids, cannabis, and other substances, excluding tobacco and opioid maintenance treatment. Three variation measures—Extreme Quotient (EQ), Coefficient of Variation (CV), and Systematic Component of Variation (SCV)—were used to assess disparities.

**Results:**

Geographic variation in SUD treatment rates ranged from 3.6 to 11.5 per 1,000 inhabitants reaching a threefold difference between areas (EQ = 3.1). We found that SCV values (8.7–23.5) and SCV _5–95_ (5.7–14.5) for diagnose groups and service type consistently exceeded the threshold of high and extremely high variation. Procurement of private services increased capacity significantly but did not markedly reduce variation. Variation remained extremely high even when the highest and lowest rates were excluded (SCV 13.8, SCV_5–95_ 11.3).

**Conclusion:**

Patient rates in SUD treatment fell every year between 2017–2021 and the geographic variation was high to extremely high. Treatment of substance use disorders in Norway may require stronger regional governance to reduce unwarranted variation and ensure equitable access to treatment. Substantial reductions in variation can be achieved by i) redistributing capacity among catchment areas, ii) purchasing fewer and shorter Inpatient stays and iii) increasing outpatient treatment. In addition, such means could dramatically increase patient rates. There is a need for more consistent clinical practices and adjusted capacity for treating specific substance diagnoses.

**Supplementary information:**

The online version contains supplementary material available at 10.1007/s43999-025-00084-y.

## Background

Worldwide there is a great impact of substance use disorders (SUDs) related to morbidity and mortality, economic costs related to care and crime and a great burden on families. Among all the risk factors associated with premature death, alcohol use and alcohol use disorder rank seventh. The contribution to premature death is partly due to the direct effects of drugs, from e.g., overdoses and partly due to long-lasting negative effects on general health [1].

Alcohol use disorder (AUD) is the most common substance use disorder (SUD) in Norway. The 12-month prevalence of harmful use or dependence on alcohol for adults is 5–8 present [2]. which means between 200–300,000 people having AUD, while the figure for SUDs is 40–70,000.

In Norway, between 2010 and 2022, the yearly number of overdose deaths was around 280, a death rate of 5.6 per 100,000 [3]. SUDs are also important risk factors for suicide. SUDs are associated with significantly increased risks, with HRs ranging from 2.5- to 6.4 [4].

Due to these high tolls of premature death, costs incurred by the society and low quality of life for patients with SUD there is a great need for evidence-based treatment. The United Nations Office on Drugs and Crime (UNODC)-WHO International Standards for the Treatment of Drug Use Disorders have set principles for the treatment system recommending that treatment services should be accessible, affordable, evidence-based, diversified with a focus on improved functioning and well-being. Further, the provision of services should be person-centred, equitable, and data-driven [5].

It is important to study any geographical variation in utilisation of health services, as large geographic differences may reveal divergence from these criteria. Very few studies have been done. One US study found that only 21% of the variations in treatment utilisation stemmed from the prevalence of SUDs and related problems [6]While another found that access to treatment was related to insurance [7].

Geographical variation in SUD treatment is thus under-researched, and Norway may be a suitable case as treatment for substance abuse in Norway is primarily publicly funded. Most in-services are free of charge for residents while visiting a GP and outpatient treatments require co-payments. Treatment typically involves collaboration between various healthcare providers, including general practitioners (GPs), specialists in addiction medicine, psychologists, social workers, and welfare services [8].

Outpatient treatment consists of psychosocial support from mental health and addiction teams, often provided in the community. There are also low-threshold facilities such as food distribution, places to sleep, and programmes that integrate substance abuse treatment with broader community services, including housing support, employment training, and social reintegration. In larger cities, there are supervised drug consumption rooms and needle exchange programmes [9].

The most common forms of treatment, where patients visit specialist clinics or healthcare centres are counselling, psychotherapy, psychoeducation, family support and peer support groups. For opioid users, outpatient clinics provide different kinds of medical maintenance therapy. Inpatient hospitals are usually long-term (weeks to months) and focus on intensive therapeutic support, addiction counselling, and psychiatric care. Detoxification, particularly for alcohol or opioid dependence, is often done in hospital settings, where patients are monitored for withdrawal symptoms [10].

Contrary to other medical and psychiatric public funded specialist services, the field of substance abuse has a slightly different structure. More than 60% of the institutions providing in-service hospitalisation are private, non-profit (religious and ideological) institutions [11]. These differences have deep historical roots and there has been relatively large political agreement that the private sector should have such a large proportion of long-term Inpatient care.

In Norway, specialised healthcare services are publicly funded and free at the point of use, with an annual out-of-pocket maximum fee limited to 3165 NOK (≈ €270 in 2024). Services are financed through general taxation, and specialised substance abuse treatment is provided by hospitals, community health centres, and private facilities contracted by the four Regional Health Authorities (RHAs). The RHAs operate through 21 Healthcare Trusts that are responsible for delivering specialised services within defined catchment areas [12, 13]. Patients have the right to choose among providers within or across regions, although cross-regional treatment is subject to capacity constraints [14].

Due to substantial differences in how health and social services are organised across Norwegian counties—including geographic variation in proximity to clinical services and a strong reliance on private institutions—there is a considerable risk of uneven access to substance abuse treatment depending on where individuals live. Norway currently has a national risk adjusted finance model for regions but lacks one national model for hospital trusts’ catchment areas. Consequently, it remains unclear whether observed differences in service utilisation reflect true variation in need or inequities in access.

While our research group recently described geographical variation in mental healthcare services in Norway [15], similar analyses for substance abuse treatment are scarce. Research on geographical variation in healthcare utilisation, inspired by the pioneering work of Wennberg and Gittelsohn [16], has mainly focused on somatic and mental health services, leaving a notable gap concerning substance abuse care. Moreover, few studies have examined both inpatient and outpatient substance abuse services to understand whether geographic variation manifests similarly across care levels.

Therefore, this study aims to describe geographic variations in patient and service utilisation rates for substance use disorders—both overall and across four major substance categories—by analysing inpatient and outpatient care separately. This will provide a more comprehensive picture of regional differences in the use of substance abuse services in Norway.

## Methods

### Study design and setting

This study is a *nation-wide registry-based cross-sectional study* of the use of specialised substance use disorder (SUD) health services in Norway from 2017 to 2021. We followed the *Strengthening the Reporting of Observational Studies in Epidemiology (STROBE)* guidelines to ensure transparent and complete reporting [17].

Specialised substance abuse treatment in Norway is delivered within the publicly funded specialist health service, organized under four Regional Health Authorities (RHAs) and their 21 Healthcare Trusts. Each Healthcare Trust is responsible for a defined hospital catchment (referral) area. However, patients may seek care across regions and catchment areas. The chois to travel over regions is limited by service capacity, but choosing a provider in another catchment area, within own region, is a patient’s legal right. Reimbursement will follow patients to the chosen provider and travel costs will be compensated. This policy empowers patient choice, reduces potential access barriers, creates a certain competition between providers.

This health atlas study reports results based on patients’ residential area, corresponding to the 21 health trusts catchment area. Patients who received treatment outside their own catchment area were still attributed to their residential area in all analyses, ensuring that the reported variation reflects population-based service use rather than the location or capacity of service providers.

### Data source: the Norwegian patient Registry (NPR)

Data for this study were obtained from the Norwegian Patient Registry (NPR). The NPR is an administrative database managed by the Norwegian Directorate of Health, which collects individual-level data on all contacts with publicly funded specialist healthcare services in Norway. Reporting to the NPR is mandatory for all public hospitals and private institutions contracted by RHAs, ensuring complete national coverage [18, 19].

The registry includes detailed information on patient demographics (age, sex, and residential area), diagnostic codes (ICD-10) and treatment activity. NPR is widely used in research on healthcare utilisation and quality of care [20–22]. The NPR data are therefore regarded as representative of the Norwegian population and suitable for studying geographical variations in health service utilisation.

### Study population

The study included all adult patients who received treatment for mental and behavioural disorders due to psychoactive substance use (ICD-10 F1x diagnoses, covering F10–F19) during the study period. Inclusion and exclusion criteria were as follows: On the timestamp of episode adults (≥18 years) with an ICD-10 F1x diagnosis were included, while children and adolescents ( < 18 years) were excluded from all analyses.

Episodes coded as opioid maintenance treatment (OMT; F11.22) were excluded, as this code pertains to the distribution of medications often without active clinical consultation. Diagnoses related to tobacco use (F17) were also excluded due to few patients. Patients without a valid residential address or with an indeterminate catchment area were excluded from all analyses to ensure that rates reflected residents of defined hospital referral areas.

In total, 58,889 unique patients were identified between 2017 and 2021, with a mean age of 41 years (SD = 14.6; range: 18–96). Men accounted for 69% of the study population. Both inpatient and outpatient treatment episodes were included. The data were stratified by organizational factors (facility type, contract type, service type) and clinical/demographic factors (age, sex, and primary diagnosis).

### Study variables

#### Primary variables

The primary variables in this study were four rate-based measures of substance use disorder (SUD) treatment activity, reflecting both patient-based and service-based utilisation:

1. Outpatient patient – unique patients receiving outpatient treatment.
2. Inpatient patient – unique patients receiving inpatient treatment.
3. Outpatient contact – total number of outpatient consultations
4. Inpatient admission – total number of inpatient admissions

All four measures were calculated in the same manner, as annual averages per 1,000 person-years for the period 2017–2021. The term *patient-years* refers to the cumulative number of patients observed in each catchment area over the five-year study period. These totals were used to calculate annualised rates per 1,000 person-years, enabling direct comparison between catchment areas with differing population sizes of the same age group. The numerator represented the sum of treated patients, consultations, or admissions across the five-year period. The denominator represented the sum of catchment-area populations (≥18 years) as of January 1 each year, corresponding to the total person-years over the study period. Rates were calculated separately for all substance use disorders combined and for each of the four diagnostic categories (alcohol, opioids, cannabis, and other substances).

#### Secondary variables

Secondary variables included demographic and organizational characteristics potentially associated with variation in service utilisation:

- Sex (male, female)
- Age (in years given as continuous and categorised in groups)
- Primary diagnosis (ICD-10 F10–F19)
- Service type (inpatient or outpatient)
- Facility contract type (3): 1 public; 2 private non-profit with assignment contract; 3 private non-profit or for-profit with commission contract. Facilities with commission contracts do not have their own catchment area responsibility. Their service provision to regions occurs through procurement contracts established by the respective Regional Health Authorities.
- Catchment area corresponding to one population residential area each for the 21 hospital trusts.

#### Substance categories

Substance use disorders were grouped into four diagnostic categories based on ICD-10 codes:

1. Alcohol use disorders (F10)
2. Opioid use disorders (F11, excluding OMT F11.22)
3. Cannabis use disorders (F12)
4. Other substance uses disorders, including sedatives/hypnotics/anxiolytics (F13), cocaine (F14), stimulants including caffeine (F15), hallucinogens (F16), inhalants (F18), and psychoactive substances not elsewhere classified (F19).

## Facility classification

Hospital-trust catchment areas were used as the unit of analysis. Facilities were grouped according to contract category. Because facilities in categories 1 and 2 operate under long-term contracts and have defined catchment areas, they were analysed together when assessing the effect of capacity procurement from private-sector providers (category 3).

To preserve anonymity, facility names were removed for institutions with fewer than 30 contacts per year, and aggregated cells with fewer than 10 patients in the descriptive facility-level dataset were replaced with a blank placeholder (“–”). These anonymisation procedures applied only to the supplementary dataset listing individual institutions (Supplementary Table S1) and affected approximately 0.2% of all admissions, inpatient bed-days, and outpatient consultations. The analytical dataset used for calculating rates and measures of geographic variation was not modified and was based on complete aggregated data at the catchment-area level.

### Data quality and cleaning

The dataset was systematically checked for duplicate entries and invalid treatment durations. A total of 58,889 unique patients were identified during the 2017–2021 period, representing approximately 121,495 patient-years, with an annual average of 24,299 patients. Some patients appeared in multiple records across different facilities, diagnoses, service types, or years because they received treatment on several occasions. This is shown in Supplementary Table S2 to illustrate the multidimensional structure of the dataset, where the same individual may contribute to several analytical categories. These repeated records represent multiple treatment episodes by the same patients, not additional individuals.

### Statistical analysis

The analyses were conducted at two levels: (1) to describe overall geographic variation in service use, and (2) to examine the potential effect of capacity procurement from private providers. The latter was addressed through a sub-analysis comparing hospital catchment areas with and without procurement of additional private capacity. This approach allowed assessment of whether observed regional variation was related to differences in service capacity rather than population characteristics alone.

For each level of analysis, rates were calculated separately for inpatient and outpatient services and expressed as age- and sex-standardised rates using population data from Statistics Norway. The age and sex distribution of Norwegian population in 2019 was used as standard population.

Measures of geographic variation were used to quantify differences in service utilisation between catchment areas. These measures followed the same methodology as a previous study on patients with severe mental illness in Norway [15]. Three descriptive indicators were calculated: the extreme quotient (EQ), the coefficient of variation (CV), and the systematic component of variation (SCV).

The EQ was calculated as the ratio of the highest to the lowest rate across catchment areas. The CV, expressed as a percentage, was obtained by dividing the standard deviation of the rates by their overall mean [23]. The SCV was used to identify the non-random component of variation in healthcare utilisation rates [23–25]. High level of variation in SCV ( > 5) indicates substantial non-random variation.

To examine the effect of extreme values on measures of variation, we determined the EQ_5−95_ and SCV_5−95_, by excluding the catchment areas with a rate below the fifth percentile and above the 95th percentile.

To examine the influence of extreme values, we also calculated EQ₅–₉₅ and SCV₅–₉₅, excluding catchment areas below the 5th and above the 95th percentile. Following established guidelines, SCV values above 3 generally reflect differences in medical practice; values between 5 and 10 indicate high variation, and values above 10 extremely high variation [23]. SCV values were adjusted for multiple contacts and admissions [26].

All analyses were performed using RStudio 2024.04.2 + 764 (R version 4.3.2).

## Results

Between 2017 and 2021 the average coverage fell every year, from 6.12 per 1000 inhabitants in 2017 to 5.46 in 2021 (Table 1). This fall of approximately 10.8% did not vary across sex.

**Table 1:** Number of substances use disorders (SUDs) patients aged ≥18 years and patient rates in the Norwegian patient Registry, 2017–2021

Distribution by sex per population	Year					
	2017	2018	2019	2020	2021	Average
Women with substance use disorders	7,905	7,645	7,623	7,431	7,315	7,584
Men with substance use disorders	17,336	16,921	16,852	16,419	16,048	16,715
Patients with substance use disorders	25,241	24,566	24,475	23,850	23,363	24,299
Population women	2,058,731	2,077,700	2,096,737	2,116,495	2,131,054	2,096,143
Population men	2,068,535	2,088,912	2,108,967	2,132,477	2,148,625	2,109,503
Population total	4,127,266	4,166,612	4,205,704	4,248,972	4,279,679	4,205,647
Patient rate women per 1,000 population	3.84	3.68	3.64	3.51	3.43	3.62
Patient rate men per 1,000 population	8.38	8.10	7.99	7.70	7.47	7.92
Patient rate total per 1,000 population	6.12	5.90	5.82	5.61	5.46	5.78

EQ: extremal quotient; CV: Coefficient of variation; SCV: Systematic Component of Variance

**Fig. 1:**
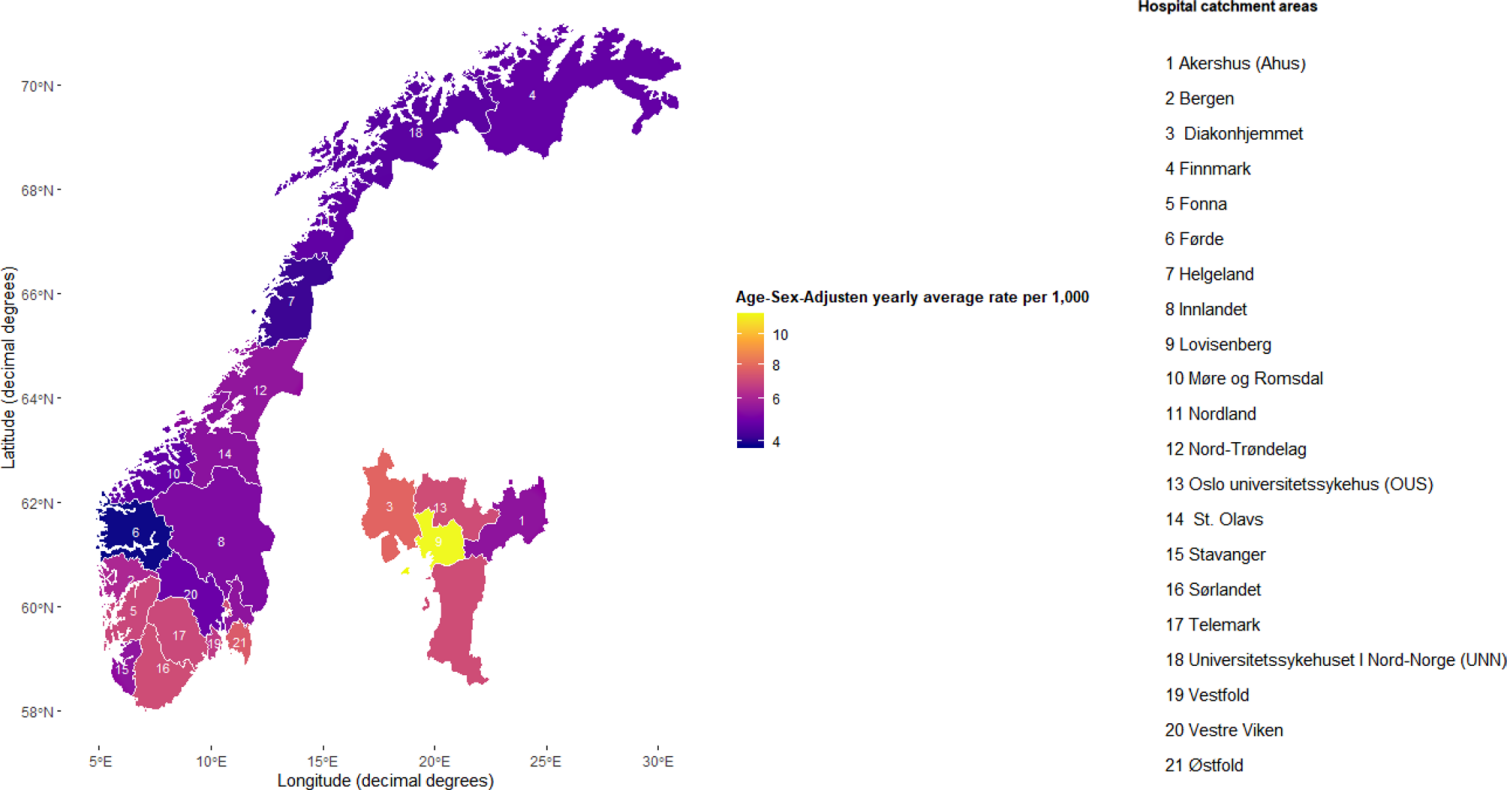
Geographic variation in substance use disorder, yearly average patient rates adjusted for age and sex in Norway for 2017–2021 in hospital catchment areas

Figure 1 and Table 2 illustrate the geographic variation in patient rates in specialised healthcare for patients with substance use disorder, by catchment areas in Norway. The coverage ranged from 3.7 per 1000 in Førde to 11.5 per 1,000 in Lovisenberg catchment area in Oslo. (Table 2) Oslo city’s four catchment areas are enlarged in the figure, due to its notable large variation within a small area (Fig. 1). The geographic variation was larger within Oslo than between other regions, most notably due to the high rate in Lovisenberg catchment area. Patient rates were higher in the south and south-west part of Norway.

**Table 2:** Average annual rates of patients with substance use disorders (SUDs) per 1000 population in Norwegian hospital catchment areas, age- and sex-standardized, 2017–2021

Catchment area	Avg. annual patients	Avg. person-years	Std. rate/1 000	95% CI low	95% CI high
Ahus	2446	442537	5,5	5,4	5,5
Bergen	2242	355644	6,2	6,0	6,2
Diakonhjemmet	924	116213	7,8	7,6	7,9
Finnmark	288	60589	4,8	4,6	4,9
Fonna	965	139840	7,0	6,8	7,1
Førde	302	85112	3,7	3,5	3,8
Helgeland	244	62546	4,2	4,0	4,3
Innlandet	1343	273427	5,2	5,1	5,3
Lovisenberg	1532	134147	11,5	11,2	11,6
Møre og Romsdal	971	208618	4,8	4,7	4,9
Nord-Trøndelag	568	106197	5,6	5,4	5,7
Nordland	504	110689	4,7	4,6	4,8
**Norway**	**24299**	**4205647**	**5,8**	**5,7**	**5,8**
OUS	1573	214274	7,2	7,0	7,2
St. Olavs	1442	262208	5,4	5,3	5,5
Stavanger	1614	280232	5,5	5,4	5,6
Sørlandet	1682	237569	7,2	7,0	7,2
Telemark	912	139038	7,0	6,8	7,1
UNN	703	154323	4,6	4,5	4,7
Vestfold	1228	194249	6,6	6,5	6,7
Vestre Viken	1827	378613	4,9	4,8	4,9
Østfold	1815	249581	7,6	7,4	7,6
**Measures of** **variation**					
Highest	11.5				
Lowest	3.7				
EQ	27.7				
EQ_95	1.9				
CV					
SCV	5.4				
SCV_95	5.0				

EQ: extremal quotient; CV: Coefficient of variation; SCV: Systematic Component of Variance, Std.rate: age and sex standardized rates

The patient rate for the whole period in Supplementary Table S2, 14.0/1,000, is smaller than the sum of diagnosis (17.3/1,000) but larger than the average patient rate of 5.8/1,000 per year in Table 1.

## There was a threefold variation between catchment areas (eq 3.1) and a CV between 25.6% and 29.8%.

More people received treatment through outpatient consultations than through inpatient admissions (Table 3). Alcohol-related disorders were the most common diagnosis in both care settings, followed by opioid and cannabis use disorders.

**Table 3:** Overall patient rates and measures of geographic variation in sud patient rates in Norway 2017–2021, from outpatient consultations and admissions, within four substance groups

Measures	Service type									
	Outpatient consultations					Admissions				
	**Any substance**	**Alcohol**	**Opioid**	**Cannabis**	**other substances**	**Any substance**	**Alcohol**	**Opioid**	**Cannabis**	**Other substances**
Average rates	5.8	5.1	2.4	0.7	0.9	1.5	2.1	1.0	0.3	0.2
**Measures of variation**										
EQ	3.3	4.0	7.9	5.0	3.1	2.3	2.8	5.7	5.0	2.8
EQ_5−95_	2.3	2.8	2.9	3.8	2.5	1.6	1.8	3.7	2.8	2.1
CV	33.3	41.3	41.4	40.4	34.4	23.6	29.9	41.6	39.6	29.3
SCV	8.6	12.5	13.2	14.7	10.5	3.0	5.5	10.6	12.0	27.0
SCV_5−95_	6.3	7.8	8.2	11.6	8.6	1.9	3.3	7.1	6.9	27.0

EQ: extremal quotient; CV: Coefficient of variation; SCV: Systematic Component of Variance

Geographic variation in treatment activity was analysed separately for outpatient consultations and inpatient admissions, as well as across the four major substance categories (alcohol, opioids, cannabis, and other substances). Overall, geographic variation was markedly higher for outpatient consultations compared to inpatient admissions. The age- and sex-standardized outpatient consultation patient rates varied more than threefold between catchment areas (EQ = 3.3, SCV = 8.6), while inpatient admissions patient rates showed smaller but still notable variation (EQ = 2.3, SCV = 3.0).

Among substance groups, the greatest variability was observed for cannabis and opioid-related disorders, particularly in outpatient care (SCV = 14.7 and 13.2, respectively). In contrast, alcohol-related disorders showed relatively lower variation in inpatient settings (SCV = 5.5).

Concerning substance group differences, the EQ was higher for specific substance groups in outpatient consultations, such as opioids (7.9) and higher for opioids and cannabis in admissions (5.7 and 5.0), suggesting particularly pronounced disparities in these categories. The two other variation measures, (CV and SCV), showed the highest heterogeneity for opioids, with CV values reaching 41.4% for outpatient consultations and 41.6% for admissions. SCV values exceeding 10 (range 10.6–27.0) for all substances but alcohol (5.5), in consultations and admissions.

We found (Supplementary Table S3) that rates for outpatient consultations (EQ 4.5–10.1), admissions (EQ 2.6–6.1), and bed-days (EQ 3.2–7.9) varied widely. The most extreme variation was found in opioid outpatient consultations, with a 23-fold EQ. We found that SCV values (8.7–23.5) and SCV _5–95_ (5.7–14.5) for diagnose groups and service type consistently exceeded the threshold of high and extremely high variation [15, 23].

Regional Health authorities (RHA) operate with two contract types: assignment and procurement to establish the desirable capacity in regions. The effect on geographic variation by the two contract types is shown in Table 4. Negative numbers in variation for procured capacity reduces variation in the total capacity. Overall, for Norway, procurement capacity increased outpatient consultations rates from 51.3 to 61.4 and admission rates from 2.9 to 3.9. All measures of variation in procured outpatient consultation were higher than for admissions. The procurement of services increased EQ, EQ_5–95_ an CV but slightly reduced the SCV and SCV _5–95_ in outpatient treatment provision.

**Table 4:** Effect of procurement on geographic variation in utilisation rates of sud outpatient consultations, admissions and bed-days, in Norway 2017–2021 per 1000 population in the catchment area adjusted for age and sex

				**Service type**					
	**Outpatient consultations**			**Admissions**			**Bed-days**		
	**Assignment**	**Total capacity**	**Effect of procurement**	**Assignment**	**Total capacity**	**Effect of procurement**	**Assignment**	**Total capacity**	**Effect of procurement**
**Catchment area**									
Ahus	51.30	59.60	8.40	2.50	3.20	0.70	42.40	119.00	76.60
Bergen	69.20	73.40	4.20	3.40	3.90	0.40	95.40	123.70	28.30
Diakonhjemmet	67.60	88.50	20.90	2.60	3.50	0.90	34.00	120.70	86.80
Finnmark	18.00	18.70	0.70	3.60	4.50	0.90	100.00	178.70	78.60
Fonna	31.50	82.20	50.80	2.40	3.50	1.10	68.50	118.90	50.40
Førde	25.50	26.30	0.80	2.00	2.60	0.60	55.80	81.30	25.50
Helgeland	26.60	27.20	0.60	1.80	2.90	1.10	37.70	118.20	80.50
Innlandet	49.30	55.00	5.70	1.90	2.90	1.00	39.00	148.60	109.60
Lovisenberg	69.70	116.50	46.80	5.10	6.60	1.50	55.20	221.50	166.30
Møre og Romsdal	51.70	54.60	2.90	4.10	4.60	0.40	85.50	123.60	38.20
Nordland	25.60	28.70	3.10	2.80	4.10	1.30	63.30	158.10	94.80
Nord-Trøndelag	44.60	53.90	9.20	2.10	3.40	1.30	50.80	127.90	77.20
**Norway**	51.30	61.40	10.10	2.90	3.90	1.00	57.70	132.10	74.40
OUS	50.20	76.30	26.20	3.40	4.40	1.00	43.40	146.40	103.00
St. Olavs	55.00	77.40	22.40	2.50	4.10	1.60	35.30	96.20	60.90
Stavanger	36.70	62.10	25.40	1.60	3.50	2.00	53.70	135.50	81.80
Sørlandet	90.60	93.00	2.40	3.80	4.20	0.50	83.10	146.60	63.50
Telemark	48.10	65.90	17.80	3.10	4.90	1.80	34.70	225.30	190.50
UNN	35.80	37.20	1.40	3.80	4.70	0.90	82.50	152.70	70.20
Vestfold	74.40	77.90	3.50	3.10	3.80	0.70	67.60	159.20	91.50
Vestre Viken	35.40	41.30	5.90	2.10	2.60	0.50	39.80	92.00	52.20
Østfold	86.30	89.60	3.30	3.20	3.80	0.60	51.00	136.70	85.60
**Measures of variation**									
EQ	5.00	6.20	1.20	3.20	2.60	−0.70	2.90	3.20	0.30
EQ_5−95_	3.40	3.60	0.20	2.30	1.90	−0.40	2.70	2.40	−0.30
CV	40.20	41.50	1.40	29.70	23.50	−6.20	35.10	26.10	−9.00
SCV	15.00	13.80	−1.20	6.90	3.20	−3.70	13.50	5.20	−8.30
SCV_5−95_	11.40	11.30	−0.10	5.80	2.20	−3.60	11.00	2.50	−8.50

EQ: extremal quotient; CV: Coefficient of variation; SCV: Systematic Component of Variance

For bed-days quite a lot of the capacity was procured and thereby increased the bed-day rates from 57.7 to 132.1. Service procurement increased EQ slightly from 2.9 to 3.2 but decreased significantly the SCV and SCV _5–95_ in admissions (6.9–3.2 and 5.8–2.2) and bed-days (13.5–5.2 and 11.0–2.5).

## Discussion

The present study found that 14 per 1000 Norwegian population had received treatment for a SUD in the five-year observation period from 2017 to 2021. The annual rate of treatment declined each year, from 6.1 to 5.5 per 1,000 residents. Some patients had several episodes, treated for more than one diagnosis and in more than one year. This was adjusted for in the analysis of variation [27].

Geographically across the country the access to and utilisation of treatment varied significantly, with over threefold differences in rates between catchment areas. Variation increased notably when analysing rates by diagnostic groups, service types, and amounts of treatment offered, including outpatient consultations or admissions, as well as Inpatient length of stay. Most variation measures showed high to extremely high variability, suggesting systematic inequalities in clinical practice and capacity [15, 23]. All analyses in this study were based strictly on patients registered place of residence, corresponding to one hospital catchment area. Patients treated outside their home region were attributed to their residential area. Consequently, the observed geographic variation cannot be explained by patients’ choice of traveling to a different catchment area or region. The findings demonstrate higher variation in access to and delivery of outpatient care for SUD compared to inpatient care, indicating possible differences in local service capacity, referral practices, and follow-up intensity between catchment areas.

Regional Health Authorities procure services from private providers to increase capacity and reduce unwarranted geographic variation in service utilisation. Procurement was more pronounced for Inpatient treatment. Twice as many bed-days were purchased from private institutions without a catchment area responsibility, compared to those offered by the governmental institutions themselves. For outpatient treatment only one in six consultations were purchased. Service procurement reduced variation in service utilisation, but the geographical variation remained high for bed-days (SCV 5.2) and extremely high consultation (SCV 13.8, SCV_5–95_ 11.3).

The overall treatment rate for SUD of 14 per 1000 residents in Norway is difficult to evaluate, as such a figure needs to be related to SUD morbidity in the country. But we know from earlier research that the coverage of AUD treatment for those in need is quite low, maybe only catching between 5 and 10% of those with alcohol use disorder [28], lower in Norway than in many European countries [29] and the rest of the world [30]. The treatment rate for other SUDs is probably higher, although it is more difficult to ascertain the prevalence of other SUDs on a population level, some sources indicate that e.g., opioid use disorders are have more than 50% coverage [31]. More dramatic is finding a falling rate of treatment in the face of increasing drug problems and clear governmental intentions of increasing the treatment rates [32]. The two last observation years were during the COVID-19 pandemic, a time of great challenge for many SUD patients [33], but the downward trend started before the pandemic years and cannot be attributed this.

The large variation in services utilisation across the country is so substantial that it cannot be attributed merely to chance. Studies suggest that differences in socio-demographics factors and morbidity explain only a small part of these geographic variations, with service availability playing a much larger role [34]. Nevertheless, systematic factors could also contribute to the observed heterogeneity. For example, differences in how hospital catchment areas are drawn, inter-catchment patient flows, and regional variations in data reporting practices might affect the rates.

While this study did not directly investigate explanatory factors, several plausible mechanisms may account for the observed geographical variation. Previous research on mental health and addiction services in Norway suggests that regional disparities in service organization, such as differences in treatment capacity, procurement practices, and referral pathways, can substantially influence utilisation rates [15, 18, 35]. Population factors—including socioeconomic disadvantage, local substance use patterns, and differences in help-seeking behaviour—are also likely to contribute [36–38]. Conversely, lower rates in rural or remote areas may reflect barriers to access, such as travel distance or limited availability of specialised services, rather than lower treatment need [39]. In addition, systematic factors related to data structure, such as inter-catchment patient flows or variation in registry reporting, might explain some of the observed heterogeneity. Together, these potential explanations suggest that the geographical variation reflects structural and organizational differences in care provision rather than random variation.

The substantial variation observed within Oslo likely reflects a combination of organisational and population-related factors. The city is divided into four hospital catchment areas served by Lovisenberg Diakonale Sykehus, Diakonhjemmet Sykehus, Oslo University Hospital and Akershus University Hospital, which differ in size, service profile and population characteristics. Lovisenberg’s catchment includes inner-city boroughs, which have higher levels of social deprivation and substance-related problems, whereas Diakonhjemmet’s area covers more affluent western boroughs. According to recent local data, the proportion of low-income households ranged from around 22% in some inner-city districts to under 6% in the western part of the city [36, 37].

Local tradition and stakeholders may play a part in geographical variation, but it is important to acknowledge that such variation in general can indicate both over- and undertreatment of health problems [40]. However, if we consider the low treatment coverage rate it is reasonable to claim that we are only talking about various degrees of undertreatment. In addition to strengthening the argument on undertreatment of SUD, variation in treatment offered is itself a non-desired feature. It is a stated goal of the government to work against such variation and ensure that all citizens are provided with the same treatment opportunity regardless of where they live or seek treatment [32]. This is also underlined as a beneficial goal worldwide [41]. The present study indicates that we are very far from such a goal.

It is a tradition in Norway, as in many countries, that different idealistic and charitable NGOs provide services for people with SUD [42]. The high procurement rates found in the present study illustrates that this is very much the situation in Norway even today. Especially long-term Inpatient stays are provided by services that the government buys from NGO. This was especially true for a significant procurement of bed-day. One could expect that such a procurement would decrease the variation in patient rates and service rates to an explicable level, e.g. SCV lower than three. When high levels of geographically variation remains, within and across Norway’s four health regions, Regional Health Authorities’ governance have failed to provide equal access to SUD services.

Redistribution of treatment capacity could help reduce unwarranted variation and improve equity in access to SUD services. Our findings indicate that areas with higher inpatient capacity tend to have longer average stays and fewer outpatient contacts, suggesting a structural imbalance between intensive and community-based care. By reallocating some inpatient resources toward outpatient and day-based services, Regional Health Authorities could reach more patients with the same or lower resource use, Such redistribution would also enhance continuity of care and support the ongoing shift toward person-centred, recovery-oriented services, as emphasized in current national strategies [35, 43].

## Strengths and limitations

The current study is based on data from the Norwegian Patient Registry (NPR), which covers all specialist treatments financed by the public healthcare system. For nearly all specialised health services, this represents close to complete national coverage. However, there is a small market for private insurance or out-of-pocket payment in SUD treatment, as in somatic healthcare. Since this market is not reimbursed by the government they do not report claim data to the NPR [35, 44]. These facilities constitute only a marginal share of the total treatment capacity in Norway since services have a global coverage. [45].

Similarly, the current study did not include SUD treatment provided in primary care or through municipal low-threshold services, such as general practitioners, outreach teams, or social care facilities. While these sectors contribute to the continuum of care. [43]. We know from earlier studies that most diagnoses of SUD—approximately 80–85%—are recorded in the specialised healthcare system [28].

Furthermore, the diagnoses used are set by the clinicians in their daily practice and are not quality controlled by others. This may lead to diagnostic inaccuracy, but we have no reason to believe that this inaccuracy varies across the investigated catchment areas. Estimating treatment need across catchment areas has also proven difficult without a national risk adjusted finance model. Future studies that incorporate individual-level socioeconomic factors or ecological designs may further clarify the observed variation.

In the Norwegian specialist health service, facilities providing SUD treatment operate under two main contractual models: assignment contracts and commission (procurement) contracts. Institutions with assignment contracts—primarily public and private non-profit—carry long-term responsibility for the population in a defined catchment area and are integrated into regional capacity planning. In contrast, facilities with commission contracts are procured by Regional Health Authorities to provide short-term capacity, often to reduce waiting times or deliver specific treatment types. This procurement mechanism enhances flexibility but may also contribute to regional differences in the availability and continuity of care, as procured capacity varies between contract periods [44]. Differences in treatment capacity across catchment areas were not assessed, as such data were unavailable and beyond the scope of this study. Nevertheless, variation in service capacity between areas may contribute to some of the geographic variation observed.

## Conclusion

Patient rates in SUD treatment fell every year between 2017–2021 and the geographic variation was high to extremely high. Treatment of substance use disorders in Norway may require stronger regional governance to reduce unwarranted variation and ensure equitable access to treatment. Substantial reductions in variation can be achieved by i) redistributing capacity among catchment areas, ii) purchasing fewer and shorter Inpatient stays and iii) increasing outpatient treatment. In addition, such means could dramatically increase patient rates. There is a need for more consistent clinical practices and adjusted capacity for treating specific substance diagnoses.

## Disclaimer

Data from the Norwegian Patient Registry has been used in this publication. The interpretation and reporting of these data are the sole responsibility of the authors, and no endorsement by the Norwegian Patient Registry is intended or should be inferred.

## Electronic supplementary material

Below is the link to the electronic supplementary material.

Supplementary Material 1

Supplementary Material 2

Supplementary Material 3

## Data Availability

The data underlying this article cannot be shared publicly due to legal restrictions.
