# Supplementary material for: Geographic variation in the utilisation of specialist healthcare for patients with substance use disorders in Norway: a population-based registry study
Source: Res Health Serv Reg. 2026 Jan 5;5:1. doi: 10.1007/s43999-025-00084-y (PMC12770024; doi:10.1007/s43999-025-00084-y)
Supplement: Supplementary file 1 — Supplementary Material 1 [file 43999_2025_84_MOESM1_ESM.docx]

| Measures | Types of substance use disorder | | | | |
| --- | --- | --- | --- | --- | --- |
|  | Alcohol | Opioid | Cannabis | Other drugs | Total (any SUD) |
| Unique patients | 30,855 | 8,534 | 11,902 | 21,324 | 58,889 |
| Duplicate patients (patient with several episodes) | 58,633 | 16,415 | 21,065 | 40,190 | 136,303 |
| Out-patient consultations | 608,395 | 134,010 | 227,542 | 393,400 | 1,363,347 |
| Unique outpatients | 27,530 | 7,394 | 10,612 | 17,872 | 53,648 |
| Admissions | 37,350 | 8,308 | 6,662 | 25,950 | 78,270 |
| Unique admitted patients | 13,807 | 4,104 | 3,927 | 11,009 | 27,832 |
| Bed-days | 1,125,130 | 289,885 | 355,656 | 1,003,655 | 2,774,326 |
| Out-patient consultations per unique out-patient | 22.1 | 18.1 | 21.4 | 22.0 | 25.4 |
| Admissions per unique admitted patient | 2.7 | 2.0 | 1.7 | 2.4 | 2.8 |
| Bed-days per unique admitted patient | 81.5 | 70.6 | 90.6 | 91.2 | 99.7 |
| Population per year (average 2017–2021) | 4,205,647 | 4,205,647 | 4,205,647 | 4,205,647 | 4,205,647 |
| Patient crude rates per 1,000 adult population | 7.34 | 2.03 | 2.83 | 5.07 | 14.00 |

Supplementary Table S2. Number of unique substance use disorders (SUDs) patients aged ≥18 years in diagnostic groups according to ICD-10 and their distribution across service types. Data from the Norwegian Patient Registry, 2017–2021.

SUD; Substance use disorder
