# Supplementary material for: Geographic variation in the utilisation of specialist healthcare for patients with substance use disorders in Norway: a population-based registry study
Source: Res Health Serv Reg. 2026 Jan 5;5:1. doi: 10.1007/s43999-025-00084-y (PMC12770024; doi:10.1007/s43999-025-00084-y)
Supplement: Supplementary file 3 — Supplementary Material 3 [file 43999_2025_84_MOESM3_ESM.docx]

**Supplementary table S3** Geographic variation in substance groups utilization rates of out-patient consultations, admissions and bed-days, in Norway 2017-2021, per 1,000 population in the catchment area adjusted for age and sex.

|  | **Service type** | | | | | | | | | | | | | | |
| --- | --- | --- | --- | --- | --- | --- | --- | --- | --- | --- | --- | --- | --- | --- | --- |
|  | **Out-patient consultations** | | | | | **Admissions** | | | | | **Bed-days** | | | | |
|  | **All substances** | **Alcohol** | **Opioid** | **Cannabis** | **All other substances** | **All substances** | **Alcohol** | **Opioid** | **Cannabis** | **All other substances** | **All substances** | **Alcohol** | **Opioid** | **Cannabis** | **All other substances** |
| **Catchment area** | | |  |  |  |  |  |  |  |  |  |  |  |  |  |
| Ahus | 59.6 | 28.1 | 7.1 | 9.9 | 14.5 | 3.2 | 1.6 | 0.5 | 0.2 | 0.9 | 119.0 | 49.5 | 17.2 | 13.0 | 39.3 |
| Bergen | 73.4 | 31.7 | 6.2 | 13.4 | 22.1 | 3.9 | 1.6 | 0.4 | 0.3 | 1.6 | 123.7 | 46.5 | 13.7 | 13.5 | 50.1 |
| Diakonhjemmet | 88.5 | 46.5 | 5.9 | 11.5 | 24.7 | 3.5 | 2.1 | 0.4 | 0.2 | 0.9 | 120.7 | 65.4 | 11.1 | 9.3 | 34.9 |
| Finnmark | 18.7 | 9.0 | 0.5 | 2.3 | 6.9 | 4.5 | 2.3 | 0.2 | 0.2 | 1.7 | 178.7 | 87.8 | 7.5 | 11.8 | 71.6 |
| Fonna | 82.2 | 34.4 | 8.8 | 10.9 | 28.1 | 3.5 | 1.3 | 0.4 | 0.3 | 1.5 | 118.9 | 32.4 | 14.1 | 14.8 | 57.5 |
| Førde | 26.3 | 14.7 | 2.5 | 2.7 | 6.4 | 2.6 | 1.5 | 0.1 | 0.1 | 0.8 | 81.3 | 47.2 | 5.2 | 5.4 | 23.5 |
| Helgeland | 27.2 | 11.2 | 5.1 | 2.8 | 8.1 | 2.9 | 1.6 | 0.3 | 0.2 | 0.8 | 118.2 | 60.0 | 8.5 | 13.6 | 36.0 |
| Innlandet | 55.0 | 22.2 | 7.6 | 10.4 | 14.8 | 2.9 | 1.4 | 0.3 | 0.3 | 0.9 | 148.6 | 58.5 | 13.5 | 26.6 | 49.9 |
| Lovisenberg | 116.5 | 68.8 | 9.1 | 15.1 | 23.5 | 6.6 | 3.8 | 0.9 | 0.3 | 1.6 | 221.5 | 110.9 | 35.0 | 16.4 | 59.1 |
| Møre og Romsdal | 54.6 | 21.7 | 5.2 | 6.3 | 21.4 | 4.6 | 2.6 | 0.2 | 0.4 | 1.4 | 123.6 | 54.6 | 8.4 | 19.9 | 40.8 |
| Nordland | 28.7 | 12.8 | 2.5 | 3.8 | 9.5 | 4.1 | 2.2 | 0.4 | 0.3 | 1.3 | 158.1 | 77.8 | 15.7 | 14.5 | 50.1 |
| Nord-Trøndelag | 53.9 | 28.2 | 3.1 | 9.9 | 12.6 | 3.4 | 1.6 | 0.2 | 0.5 | 1.1 | 127.9 | 49.2 | 8.2 | 27.1 | 43.4 |
| **Norway** | **61.4** | **28.0** | **6.0** | **9.8** | **17.7** | **3.9** | **1.9** | **0.4** | **0.3** | **1.3** | **132.1** | **53.6** | **13.8** | **16.9** | **47.8** |
| OUS | 76.3 | 44.6 | 5.9 | 11.2 | 14.6 | 4.4 | 2.7 | 0.7 | 0.2 | 0.8 | 146.4 | 72.2 | 24.5 | 16.4 | 33.3 |
| St. Olavs | 77.4 | 38.2 | 11.6 | 10.7 | 16.8 | 4.1 | 2.3 | 0.4 | 0.3 | 1.1 | 96.2 | 43.9 | 10.5 | 13.8 | 28.0 |
| Stavanger | 62.1 | 21.1 | 3.6 | 11.0 | 26.4 | 3.5 | 1.4 | 0.3 | 0.3 | 1.5 | 135.5 | 43.6 | 9.0 | 14.3 | 68.6 |
| Sørlandet | 93.0 | 33.5 | 6.9 | 23.7 | 28.9 | 4.2 | 1.6 | 0.4 | 0.6 | 1.6 | 146.6 | 54.2 | 10.7 | 25.7 | 56.0 |
| Telemark | 65.9 | 20.5 | 7.5 | 14.9 | 22.9 | 4.9 | 1.8 | 0.5 | 0.7 | 1.9 | 225.3 | 73.5 | 16.3 | 40.6 | 94.8 |
| UNN | 37.2 | 16.1 | 6.9 | 3.3 | 10.8 | 4.7 | 2.5 | 0.5 | 0.3 | 1.4 | 152.7 | 63.5 | 15.8 | 15.3 | 58.1 |
| Vestfold | 77.9 | 35.0 | 6.2 | 12.9 | 23.9 | 3.8 | 1.6 | 0.5 | 0.4 | 1.3 | 159.2 | 57.1 | 16.9 | 23.4 | 61.8 |
| Vestre Viken | 41.3 | 20.2 | 5.2 | 6.1 | 9.8 | 2.6 | 1.2 | 0.4 | 0.2 | 0.8 | 92.0 | 41.2 | 12.1 | 9.4 | 29.3 |
| Østfold | 89.6 | 37.4 | 8.5 | 14.6 | 29.1 | 3.8 | 1.3 | 0.3 | 0.3 | 1.9 | 136.7 | 41.0 | 12.1 | 20.0 | 63.7 |
| **Measures of variation** | | |  |  |  |  |  |  |  |  |  |  |  |  |  |
| EQ | 6.2 | 7.7 | 23.3 | 10.1 | 4.5 | 2.6 | 3.1 | 6.1 | 6.0 | 2.5 | 3.2 | 3.4 | 7.8 | 7.9 | 5.0 |
| EQ_5−95_ | 3.6 | 4.1 | 3.6 | 5.6 | 4.1 | 1.9 | 2.1 | 4.0 | 3.2 | 2.3 | 2.4 | 2.1 | 3.3 | 2.9 | 2.6 |
| CV | 41.5 | 50.0 | 43.1 | 53.6 | 43.0 | 23.5 | 32.5 | 43.7 | 42.8 | 29.5 | 26.1 | 31.2 | 48.0 | 45.3 | 34.5 |
| SCV | 13.8 | 18.6 | 16.4 | 23.5 | 16.8 | 3.2 | 8.7 | 11.7 | 15.0 | 7.6 | 5.2 | 8.7 | 14.0 | 17.5 | 11.3 |
| SCV_5−95_ | 11.3 | 13.2 | 8.3 | 15.6 | 14.5 | 2.2 | 5.8 | 7.2 | 7.8 | 5.9 | 2.5 | 5.7 | 8.4 | 9.5 | 6.8 |

EQ: extremal quotient; CV: Coefficient of variation; SCV: Systematic Component of Variance
